# Symmetrical intertriginous and flexural exanthema related to the use of paracetamol^☆^^☆☆^

**DOI:** 10.1016/j.abd.2020.08.023

**Published:** 2021-07-15

**Authors:** Joana Alexandria Ferreira Dias, Luana Moraes Campos, Juliano Vilaverde Schmitt, Sílvio Alencar Marques

**Affiliations:** Faculty of Medicine, Universidade Estadual Paulista, Botucatu, SP, Brazil

Dear Editor,

In 1984, Andersen et al.^1^ reported three patients who developed erythematous lesions on the gluteal region, inner thighs and armpits, without accompanying overall symptoms and who were systemically exposed to allergens to which they had previous contact sensitization. By analogy of the lesions to the gluteal morphological aspect of baboons (*Papio papio*), the name Baboon syndrome (BS) was suggested for these cases.^1^ Hausermann et al., in 2004, proposed the designation symmetrical drug-related intertriginous and flexural exanthema (SDRIFE) for cases of systemic drug sensitization, without topical exposure, clinically identical to BS, and highlighted the absence of accompanying systemic signs and symptoms.^2^

This is the report of a 56-year-old male patient complaining of lesions on the inguinal and axillary regions that were slightly pruritic and not associated with other systemic symptoms. He reported a two-day use of paracetamol, exclusively, and a condition identical to the current one in previous exposures. Upon physical examination, well-delimited, erythematous, wine-colored lesions were observed, without signs of excoriation, with a bilateral and symmetrical distribution on the axillary, inguinocrural, gluteal regions, and lateral aspect of the thighs, including the popliteal region (Figure 1, Figure 2). Histopathological examination of a biopsy in an axillary lesion showed epidermis with mild spongiosis, moderate dermal, lymphohistiocytic, perivascular infiltrate, and vasodilation. The absence of necrotic keratinocytes and pigmentary incontinence was observed, which ruled out fixed drug eruption (Fig. 3).

**Figure 1:**
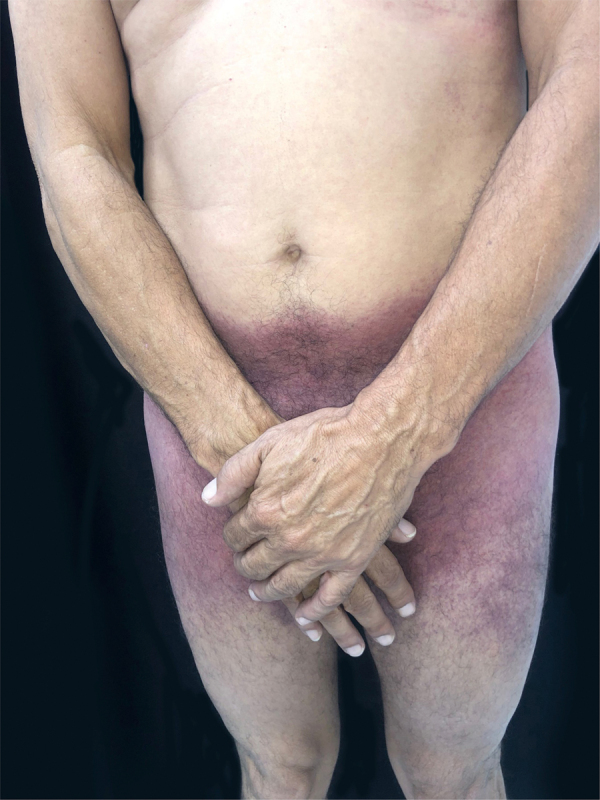
SDRIFE: erythematous-purpuric macules affecting the suprapubic region, lateral thighs and inguino-crural region.

**Figure 2:**
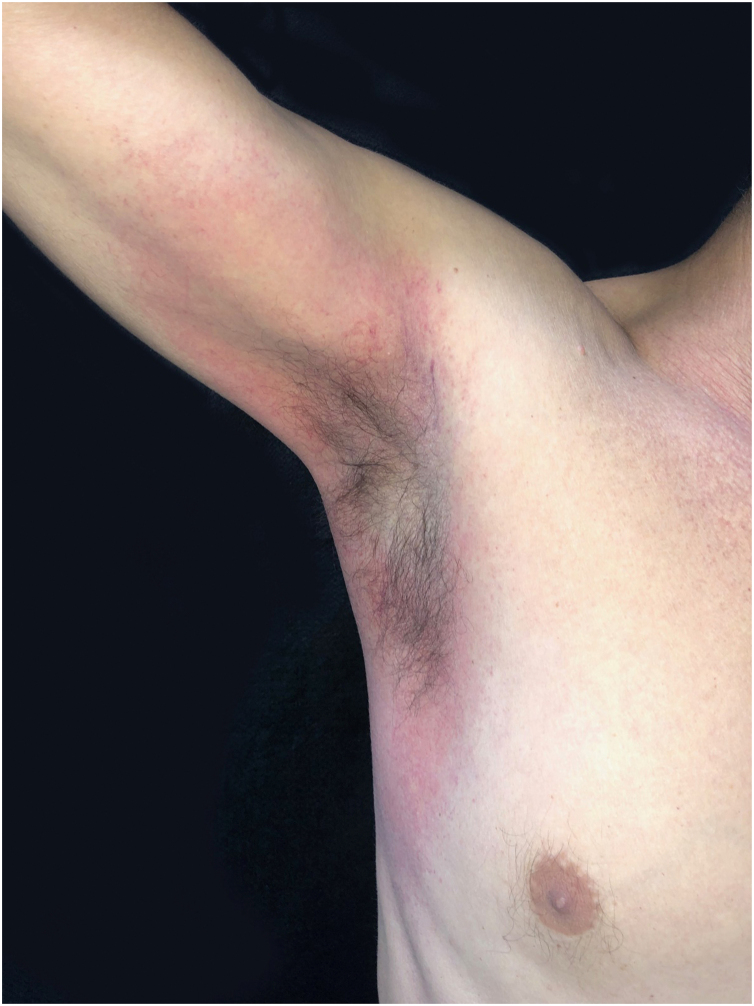
SDRIFE: evidence of axillary involvement.

**Figure 3:**
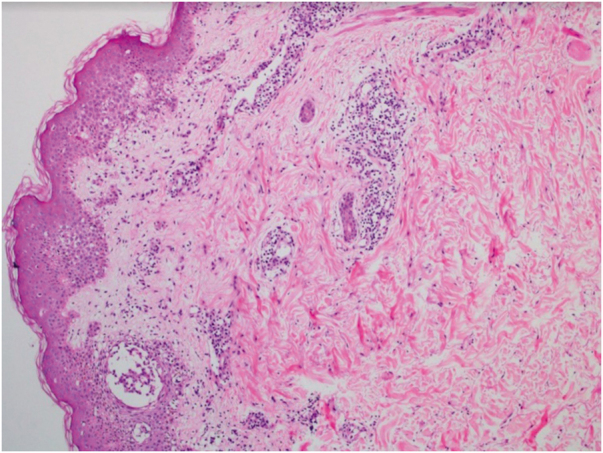
SDRIFE: Histopathology showing perivascular lymphohistiocytic infiltrate in the superficial and mid-dermis (Hematoxylin & eosin, ×100).

Therefore, the clinical, dermatological, and histopathological data allowed the diagnosis of paracetamol-induced SDRIFE. The lesions disappeared within a few weeks after drug discontinuation and the use of topical corticosteroids.

In 2011, Miyahara et al.^3^ proposed the classification of Baboon syndrome into four subtypes: 1) classic BS, resulting from sensitization by contact allergens and triggered by systemic exposure to them; 2) BS induced by contact with drugs, triggered by absorption after skin re-exposure; 3) BS induced by systemic sensitization and a condition triggered by cutaneous exposure to the drug; and 4) SDRIFE, which corresponds to systemic sensitization and manifestation when there is systemic re-exposure, excluding contact allergens.^2^, ^3^ Clinically, SDRIFE is characterized by symmetrical exanthema in the gluteal, intergluteal and inguinal area, in addition to the involvement of at least one intertriginous and flexural area such as axillary, cubital, and popliteal areas. It is considered a rare and benign manifestation of a hypersensitivity reaction with an absence of systemic symptoms. The onset occurs in hours to two days after exposure to the causative agent. The most often implicated medications are beta-lactams, particularly amoxicillin, sulfamides, anti-inflammatory drugs, barbiturates, tetracyclines, and carbamazepine. In the literature, there are only two reported cases of SDRIFE associated with the use of paracetamol.^4^, ^5^

The pathogenesis is not completely understood, but, as in allergic contact dermatitis, the picture suggests a delayed hypersensitivity reaction mediated by T cells. A greater density of eccrine sweat glands in skinfold regions would explain their restricted or predominant manifestation in intertriginous locations, where the excretion of the sensitizing drug would precipitate the dermatosis. Treatment involves suspicion and interruption of the drugs being used. Topical or systemic steroids can speed up resolution. Case reports and identification of the triggering drug are useful from a instructive and epidemiological viewpoint.

## Financial support

None declared.

## Authors’ contributions

Joana Alexandria Ferreira Dias: Approval of the final version of the manuscript; design and planning of the study; critical review of the literature.

Luana Moraes Campos: Approval of the final version of the manuscript; critical review of the manuscript.

Juliano Vilaverde Schmitt: Approval of the final version of the manuscript; collection, analysis and interpretation of data; critical review of the manuscript.

Silvio Alencar Marques: Approval of the final version of the manuscript; drafting and editing of the manuscript; critical review of the literature; critical review of the manuscript.

## Conflicts of interest

None declared.
